# Perspectives of and Experience toward the Abuse of Antidiarrheal Drug (Loperamide) among Community Pharmacists: A Cross-Sectional Study

**DOI:** 10.3390/ijerph20146400

**Published:** 2023-07-19

**Authors:** Muna Barakat, Amal Akour, Diana Malaeb, Sarah Cherri, Wala’a Al.Safadi, Ala’a Al.Safadi, Mayyada Wazaify

**Affiliations:** 1 Department of Clinical Pharmacy and Therapeutics, School of Pharmacy, Applied Science Private University, Amman 541350, Jordan; alaa.safadi9@gmail.com; 2 MEU Research Unit, Middle East University, Amman 11831, Jordan; 3 Department of Biopharmaceutics and Clinical Pharmacy, School of Pharmacy, The University of Jordan, Amman 11942, Jordan; a.akour@ju.edu.jo (A.A.); m.wazaify@ju.edu.jo (M.W.); 4 Department of Pharmacology and Therapeutics, College of Medicine and Health Sciences, United Arab Emirates University, Al Ain 15551, United Arab Emirates; 5 College of Pharmacy, Gulf Medical University, Ajman P.O. Box 4184, United Arab Emirates; dr.diana@gmu.ac.ae; 6 School of Pharmacy, Lebanese International University, Beirut 14404, Lebanon; sarashere940@gmail.com; 7 Department of Pharmaceutics & Pharmaceutical Technology, Faculty of Pharmacy, Al-Ahliyya Amman University, Amman 19328, Jordan; w.alsafadi@ammanu.edu.jo

**Keywords:** abuse, community pharmacists, Jordan, loperamide

## Abstract

This study aimed to assess the ability of community pharmacists to recognize cases of loperamide abuse at the point of sale, their perspective of and experience toward potential abuse cases. Methods: A cross-sectional study was conducted in Jordan, using a self-administered online questionnaire. The questionnaire consisted of three main parts: demographics, the experience of pharmacists with abusers’ behavior, as well as their perspectives toward loperamide abuse. Results: A total of 250 community pharmacists completed the survey, 54% (135) of which were female. Almost one-third (33.2%; 83) of the participants reported exposure to suspected cases of loperamide abuse during the last six months. Pharmacists declared that most of the suspected loperamide abusers were male (60.2%), of middle–low socioeconomic status (69.9%), and between 20 and 30 years of age (57.8%). The largest quantity (packs) of loperamide requested by a single patient was around 33.2 ± 14.9 at once. As reported by pharmacists, the suspected reasons behind loperamide abuse included 50% seeking euphoria, 17% relieving anxiety, and 33% controlling addiction (weaning off other opioids). The multivariate logistic regression analysis demonstrated a significant correlation between the male sex (OR = 1.2, 95% CI 0.12–1.59), pharmacy location in the center of Jordan (OR = 21.2, 95% CI 2.45–183.59), late-night working shift (Shift C, OR = 1.29, 95% CI 0.12–2.08), and abuse to loperamide during the last six months. Conclusions: This study sheds light on loperamide abuse potentials, which could be influenced by different sociodemographic characteristics. Accordingly, close monitoring and thorough tackling of the abuse practices are mandated through an increase in educational and awareness campaigns about proper medication use.

## 1. Introduction

Drug misuse is the use of medicine in a way inconsistent with medical guidelines, often involving prescription medication. At the same time, drug abuse is known as using prescription and over-the-counter (OTC) medicine for a nonmedical reasons to achieve euphoria [1]. The literature showed that some classes of medications, including stimulants, laxatives, sedatives, dissociative substances, opiate-containing medicines, and smoking-cessation products, can be potentially abused [2]. Owing to their accessibility and perceived safety, OTC medications tend to be abused more [2]. Loperamide, an OTC anti-diarrheal agent, acts as an agonist to mu (µ) receptors in the periphery and is expected to have low abuse potential when taken at recommended doses [3]. However, it can cross blood–brain barriers at high doses and thus cause opioid-like euphoria, leading to abuse and addiction [3,4]. Indeed, loperamide abuse and misuse cases were identified in several countries, mainly due to its low cost and ease of access [4]. Reports to the National Poison Data System in the United States between 2010 and 2015, showed that the intentional misuse, abuse, and loperamide-related suicide had increased by 91%, and among 26 cases were identified between 2011 and 2019 by the ToxIC registry, 12 (67%) were misuse/abuse cases [5,6,7] Consistently, the European Medicines Agency’s (EMA) pharmacovigilance report evaluated loperamide adverse drug reactions (ADRs) from 2005 to 2017 in the European economic areas (EAA) and non-EAA. Among 1983 ADRs, the ‘drug use disorder’ (n = 742; 37.4%), ‘intentional overdose’ (n = 502, 25.3%), and ‘intentional product misuse’ (n = 269; 14.9%) were the most common ones [2,8]. Therefore, more laboratories are including loperamide in their standard screening and clinicians should be aware of the potential abuse as it becomes increasingly pivotal to identify these potential cases of inappropriate use since loperamide overdose can result in addiction as well as serious detrimental adverse effects, such as respiratory and central nervous system depression, QTc prolongation, and even fatalities [8,9,10]. In addition to evaluating loperamide as a single substance, polysubstance abuse was also reported in up to 80% of abuse/misuse cases, with common co-exposure to antihistamines (chlorpheniramine, cimetidine), sedatives, hypnotics, and antipsychotics; antidepressants, alcohol; opioids (methadone); and cough or cold medications, such as dextromethorphan [5,6,7].

Pharmacists, accessible healthcare providers, are ideally positioned to identify abuse potential among patients. In addition, they can have a crucial role in preventing further overdoses by characterizing abuse patterns and diversion, educating about the risk of loperamide overdose, and referring risky patients to substance-use treatment [11]. In the US, an assessment of pharmacists’ awareness about loperamide abuse and their ability to restrict abuse potential is vital since around 72% of pharmacists reported their awareness about loperamide abuse and had good attitudes about their ability to decrease the quantity obtained or refused to dispense loperamide in cases of abuse potential [12]. However, only 3.2% of pharmacists had taken measures to minimize abuse potential and placed loperamide behind the counter [12]. The ability of pharmacists to identify abuse cases and how to deal with them can be affected by many factors, such as years of experience [13,14,15], which has not been widely investigated. According to a Jordanian study in 2016, and more recently in 2022, the most abused drugs in community pharmacies were decongestants, cough/cold preparations, benzodiazepines, and antibiotics [16]. However, loperamide abuse patterns in our region have not been investigated thoroughly. Therefore, this study aims to assess the ability of community pharmacists to recognize cases of loperamide abuse at the point of sale, their perspective, and experience toward potential abuse cases.

## 2. Materials and Methods

This was a cross-sectional study conducted on Jordanian pharmacists from all governorates (north, center (including the capital of Jordan), and south) from September 2021 to July 2022. The data were collected using an electronic self-administered questionnaire using a snowball convenience. In contrast to other sampling techniques, the snowball sampling process enables the researchers to sample communities that are challenging to access. The online Google Forms platform was used to run the survey, which was subsequently shared on numerous social media platforms (i.e., WhatsApp, LinkedIn, and Facebook). The survey was shared with a small pool of pharmacists (around 15–20), and each of those participants shared the link with their network of pharmacists. Those respondents then participated and shared again, and so on. There was no risk to the respondents, and their participation was anonymous and voluntary. Jordanian pharmacists and pharmacy assistants who worked in community pharmacies were eligible for study enrollment. The participant’s confidentiality was maintained throughout the data collection process. Ethical approval for the study was obtained from the Faculty of Pharmacy, Applied Science Private University (Approval number: 2021-PHA-33).

### 2.1. Study Tool

The study questionnaire was adapted from a similar published–validated tool conducted in Jordan [16,17]. The questionnaire was administered in Arabic, the native language of Jordan, and designed in Arabic. The expected filling time of the questionnaire was five minutes. About 5–20 participants per predictor are recommended as being ideal based on Tabachnick and Fidell’s recommendations for sample size calculation in the analysis [18]. Considering that the number of independent variables was 12 and the utilization of 10 individuals per predictor in this study, a minimum sample size of 120 or more was thought to be appropriate for this study’s goals.

The questionnaire was composed of three main sections: The first section covered sociodemographic characteristics of the participants (age, sex, educational level, monthly incentive status, years of experience, and working shift in the pharmacy). The second section was concerned about the pharmacy information (location, type of pharmacy, and general socioeconomic status of patients visiting the pharmacies). The third section focused on pharmacists’ experiences with loperamide abuse, such as cases of abuse, the extent of abuse, as well as characteristics of patients who seek loperamide, such as sex and socioeconomic status. Moreover, the questionnaire assessed pharmacists’ attitudes toward suspected abusers and the perceived motivation behind their abuse. At the end of the questionnaire, there was an open space for further comments that may support the aim of the study.

### 2.2. Statistical Analysis

Statistical Package for Social Sciences (SPSS) 25.0 was used to conduct the statistical analysis. The mean and standard deviations (SD) for all continuous variables were provided, and the frequencies (n) and percentages (%) of all categorical variables were used. Normality was checked using the Shapiro–Wilk test. Binary logistic regression was used to identify the factors linked with the pharmacist-reported cases of loperamide misuse. In the bivariate analysis, variables with a *p*-value of less than 0.25 were included [19]. Results were shown as odds ratios (OR) and 95% confidence intervals (CI). The two-tailed *p*-value of 0.05 was used to determine if the relationship was statistically significant.

## 3. Results

### 3.1. Sociodemographic Characteristics 

A total of 250 pharmacists completed the survey, and approximately half of them (n = 135, 54%) were female. Sixty percent (n = 150) of the respondents were less than forty years old, and the majority had a bachelor’s degree 82.8% (207). Regarding the pharmacist’s experience in community pharmacy, most participants (64%) had a monthly incentive system based on the number of medicines sold per month, and nearly half of them (47.2%) had more than ten years of experience. Most participants (74.4%) worked in pharmacies located in the middle region of Jordan, and 66% worked in pharmacies located on a main road. Based on the pharmacist perception, pharmacy customers were classified according to their socioeconomic status as low to middle or middle income (n = 110, 44%), (n = 79, 31.6%), respectively. Most participants held a “principal pharmacist” position in the community pharmacy (n = 221, 88.4%), responsible for pharmacy management and decision-making. Table 1.

### 3.2. Pharmacist Experience with Loperamide Use and Abuse

**Figure 1:**
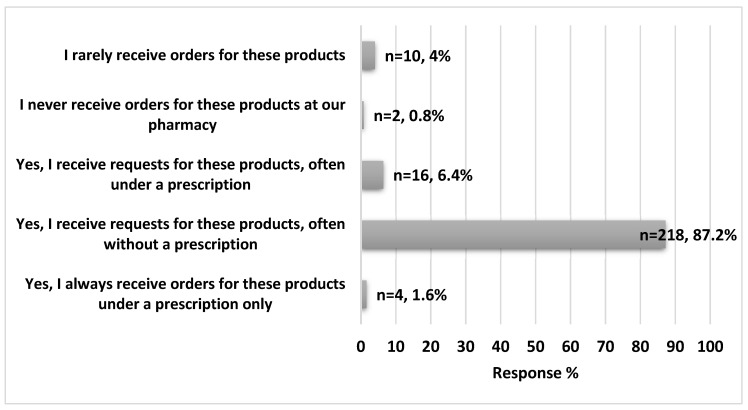
Pharmacists’ experiences with loperamide (n = 250).

The majority of the participating pharmacists received requests for loperamide use without a physician’s prescription (n = 218, 87.2%), as illustrated in Figure 1. Almost one-third (n = 83, 33.2%) of the participants reported exposure to suspected loperamide abuse cases during the last six months. Imodium^®^ caps, Loperium^®^ tab, and Imodium^®^ instant ODT were the most products increasing in improper use in Jordan (83.1%, 83.1, and 72.3%, respectively). Pharmacists declared that most of the suspected loperamide abusers were male (60.2%), of middle–low socioeconomic status (69.9%), and were between 20 and 30 years of age (57.8%), Table 2.

The majority of the respondents (85.5%) who reported the suspected loperamide abuse cases, confirmed that the suspected abuse cases are increasing with time. The largest quantity (packs) of loperamide requested from the pharmacist by a single patient was around 33 (mean 33.2 ± 14.9). The suspected abusers could be both strangers and regular patients requesting the product without a prescription (81.9%). Moreover, the suspected loperamide abusers were identified by pharmacists through some remarks, including their ability to manage mood disturbances, the ability to control impulses and thought disturbances (89.2%), exaggeration of a medical problem and/or mimicking symptoms to obtain medication (89.2%), and patients with an unusual appearance (74.7%), Table 2. 

**Figure 2:**
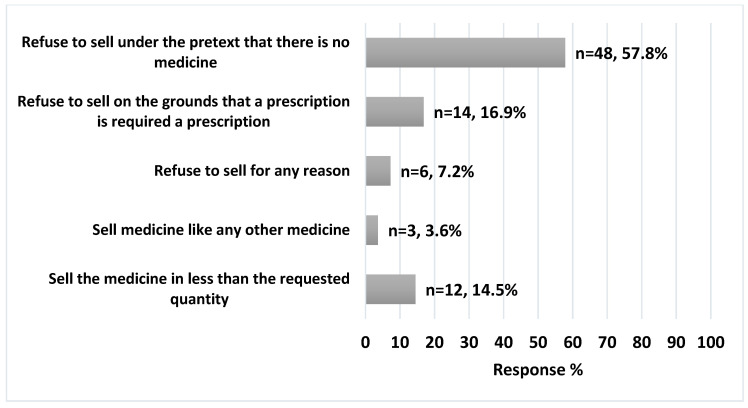
Pharmacists’ attitudes toward loperamide suspected abuse cases (n = 83).

**Figure 3:**
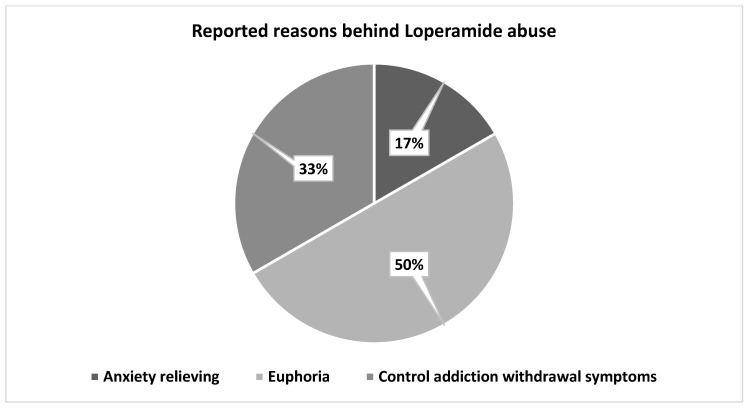
Pharmacists reported possible reasons behind loperamide abuse. This was an open-ended question, and the answers were presented in main themes.

Regarding pharmacists’ response toward suspected abusers, out of the 83 pharmacists, 57.8% (n = 48) refused to sell loperamide under the pretext of lack of medication, while 14.5% (n = 12) dispensed a quantity lower than requested, Figure 2. The pharmacist also reported that the possible reasons behind loperamide abuse included 50% seeking euphoria, 33% controlling addiction (weaning off other opioids), and 17% relieving anxiety, Figure 3.

The multivariate logistic regression analysis, Table 3, demonstrated a significant correlation between the participants’ reported loperamide abuse during the last six months and the male sex (OR = 1.2, 95% CI 0.12–1.59), pharmacy location in the center of Jordan (OR = 21.2, 95% CI 2.45–183.59), and working in the late-night shift from 12 to 8 AM (Shift C, OR = 1.29, 95% CI 0.12–2.08).

## 4. Discussion

Loperamide, a common anti-motility OTC medication, has been targeted as a drug of abuse due to its euphoric and sedating effects at supratherapeutic doses [3]. OTC drug abuse in Jordan has been documented in the last few years, with cough and cold preparations, as well as nasal decongestants, being the most reported medications [16,20]. Although there are no definitive data on the extent of loperamide abuse in Jordan, a recent study conducted by Webb et al. indicates that nonmedical use of loperamide is common in the United Kingdom (UK) and the United States of America (US) [21]. Moreover, numerous case reports, in addition to the state poison control center reports that have been published in recent years, report that loperamide abuse is a cause of concern [6]. Between 2010 and 2015, epidemiological studies carried out in the US revealed a notable rise in loperamide abuse and a significant increase in intentional exposure to loperamide, with a third of cases occurring in teenagers and young adults [4,6]. This is a global health concern, and the current study is the first to examine the perspective of Jordanian pharmacists on loperamide abuse in the community.

The study found that 87.2% of pharmacists were asked for loperamide without a prescription, which is a normal expectation as it is classified as an OTC medication. However, we believe that abusers may be shifting to OTC medications for easier access, lower suspicion, and the lack of availability of their usual drugs (especially during COVID-19 pandemic). About one-third of the community pharmacists surveyed (33.2%) in our study reported receiving suspicious requests for loperamide in the past six months, and 85.5% of pharmacists believed that the loperamide abuse is rapidly growing. The study also found that 60.2% of suspected loperamide abusers were male, which is consistent with the US national poison data system, where loperamide is more likely to be abused by male subjects [6]. In parallel, it has been documented by the United Nations’ office of drugs and crime (UNDC) that opioids worldwide are abused by males, to which loperamide belongs [22]. It is not clear yet why men have more of a tendency to develop drug abuse, but previous theories attributed that to the ease of access by male peers and the enhanced sense of masculinity [23]. However, 34.9% of pharmacists reported receiving suspicious requests from both sexes, suggesting that the sex gap may be narrowing. Internet forums discussing loperamide have become more prevalent since 2010, focusing on the drug’s euphoric effects and ability to self-treat opioid withdrawal [20,24]. The majority of the pharmacists (88%) in our study believed that the feeling of euphoria and addiction control were the main reasons for loperamide abuse [7].

Pharmacists play a crucial role in controlling OTC medication abuse by monitoring their use among specific populations. Past research has identified various measures that pharmacists usually apply to reduce the likelihood of OTC medication abuse [25]. Among the most commonly used measures by pharmacists were refraining from selling the products, concealing the problematic products, and questioning the potential abusers about their purchase [26,27]. In this study, it was demonstrated that almost 58% refrain from selling loperamide under the pretext that the medication is unavailable, which is consistent with the results from another study [16,27,28,29]. Nonetheless, Jordanian community pharmacists encounter numerous obstacles when addressing OTC medication abuse, one of the most common being “pharmacy hopping”. Therefore, additional urgent measures are indispensable for drug authorities in Jordan. To address the abuse of loperamide, the US Food and Drug Administration (FDA) has implemented multiple measures; for instance, in June 2016, the FDA issued a drug safety communication warning about the serious and fatal cardiac events associated with loperamide abuse, such as QT interval prolongation, torsade de pointes (TdP), and cardiac arrest [6,30]. Also, patients were cautioned against surpassing the prescribed or OTC doses since this could cause severe heart rhythm problems or even death. Additionally, a black box warning was added to Imodium’s package insert, which mentioned the instances of heart issues that had been reported due to the improper use of Imodium in supratherapeutic doses [6,30]. Loperamide is an established substrate of P-glycoprotein (P-gp), which efficiently effluxes loperamide and prevents access under normal doses. However, in cases of overdose, the saturated P-gp allows loperamide to be absorbed into the brain, leading to euphoric central nervous system (CNS) effects. The liver metabolizes loperamide through N-demethylation, facilitated by enzymes of the cytochrome P450 (mainly CYP2C8 and CYP3A4 [31]. The primary metabolite of loperamide, desmethylloperamide, often reaches plasma concentrations over two times higher than loperamide. At high concentrations, the metabolite acts as both a substrate and an inhibitor of P-gp, overwhelming the transporter, and has a low affinity to cardiac potassium channels in the heart. Thus, the ratio of loperamide to desmethylloperamide can provide insights into the acuteness of exposure, but the ratio may be distorted in acute or chronic administration cases. When abused, individuals frequently combine loperamide with other drugs to enhance its absorption and ability to cross the blood–brain barrier, inhibit the metabolism of loperamide, and intensify its euphoric effects [31]. Therefore, several P-gp and CYP2C8 inhibitors, such as ketoconazole, cyclosporine, cimetidine, and quinidine, can increase euphoric effects and potential toxicity from loperamide overdose [32]. 

Furthermore, the FDA approved new packaging for brand-name OTC loperamide in September 2019 to increase the safe use of loperamide products. This does not limit OTC access for consumers who intend to use loperamide appropriately [33]. The new packaging limits each package to a maximum of 48 mg of loperamide and requires unit-dose blister packaging [33]. Therefore, similar measures are warranted in Jordan to limit the cases of loperamide abuse. Also, to address this problem, drug regulatory agencies, such as the Jordan Ministry of Health (MoH) and Jordan Drug and Food Administration (JFDA), should strengthen their approach and strictly implement drug regulations, which are expected to reduce this problem. Although the 2013 Jordanian Drug and Pharmacy Practice Law requires pharmacists to keep records of prescriptions of Scheduled I–VII drugs, it does not mandate such records for other drugs, including loperamide, that are known to be abused [16]. Therefore, the law should be updated to include these medications. Another potential approach to address this issue is reclassifying medications to be misused or abused, as seen in the case of alprazolam, which was moved to a Schedule III medication by the JFDA in 2014. This change led to a reduction in access to alprazolam, which had previously been a frequently abused drug in Jordan [16]. In parallel, several studies have presented substantial proof that pharmacists can play a significant role in addressing the opioid crisis through the provision of overdose prevention training, medication reviews, and counseling [34].

There are several limitations to this study. First, response credibility may be jeopardized by knowledge bias relating to the availability of resources on demand. Second, there may be selection bias with the snowball collection method and lack of random selection. Third, possible unmeasured factors or responses to variables may be directly or indirectly related to loperamide abuse. Fourth, using an online survey rather than a face-to-face encounter risks the study data’s trustworthiness and authenticity. Fifth, the number of responses may not represent the whole study population, which raises the flag to conduct future research using a bigger sample size. Also, this study is limited by not asking about other co-abused medications.

## 5. Conclusions

In conclusion, this study sheds light on loperamide abuse potentials, which could be influenced by different sociodemographic characteristics. Each healthcare team member has a part to play, such as ensuring proper pain management, imparting education, or introducing changes at the state level. The healthcare team must act swiftly and efficiently in order to prevent harm to patients and address this growing concern.

## Figures and Tables

**Table 1:** Sociodemographic characteristics of the participants enrolled in the study (n = 250).

Variables	n (%)
**Sex**	
○ Female	135 (54.0)
○ Male	115 (46.0)
**Age**	
○ 20–30	75 (30.0)
○ 31–40	75 (30.0)
○ 41–50	70 (28.0)
○ >50	30 (12.0)
**Educational level**	
○ Diploma	31 (12.4)
○ Bachelor’s degree	207 (82.8)
○ Postgraduate degree	12 (4.8)
**Location of the pharmacy**	
○ North Jordan	35 (14.0)
○ Middle of Jordan	186 (74.4)
○ South Jordan	29 (11.6)
**Pharmacy location is in the**	
○ Main Road	165 (66.0)
○ Side Road	79 (31.6)
○ Mall	6 (2.4)
**Type of the community pharmacy**	
○ Chain pharmacy	90 (36.0)
○ Independent pharmacy	160 (64.0)
**Pharmacists working in a pharmacy have a monthly incentive system based on the quantity of medicines sold per month**	
○ No	112 (44.8)
○ Yes	138 (55.2)
**The main employment role in the pharmacy**	
○ Principal pharmacist	221 (88.4)
○ Pharmacist assistant	29 (11.6)
**Years of experience in the pharmaceutical field**	
○ <5	61 (24.4)
○ 5–10	71 (28.4)
○ >10	118 (47.2)
**Working shift at the pharmacy**	
○ First Shift (**A**, 8 a.m.–4 p.m.)	64 (25.6)
○ Second Shift (**B**, 4–12 a.m.)	21 (8.4)
○ Third Sift (**C**, 12 a.m.–8 a.m.)	70 (28.0)
○ Variable periods	95 (38.0)
**The general socioeconomic status of patients who visit the pharmacy**	
○ Low	29 (11.6)
○ Low to middle	110 (44.0)
○ Middle	79 (31.6)
○ High	32 (12.8)

**Table 2:** Pharmacists’ experiences with loperamide use and abuse.

Variables	n (%)
**During the past six months, have you had cases of suspected loperamide abuse?**	
○ Yes	83 (33.2)
○ No	156 (62.4)
○ Not sure	11 (4.4)
The products that you suspect that there is an increase in abuse in Jordan (More than one answer was allowed) *	
○ Diapen^®^ Caps	47 (56.6)
○ Imodium^®^ Caps	69 (83.1)
○ Imodium^®^ Instant ODT	60 (72.3)
○ Imotril^®^ Cap	53 (63.9)
○ Loperium^®^ Tab	69 (83.1)
○ Vacontil^®^ Tab	56 (67.5)
○ Others	2 (2.4)
In general, the socioeconomic status of the suspected loperamide abusers (for any of the products mentioned above) *	
○ Low	22 (26.5)
○ Low–middle	58 (69.9)
○ Middle	3 (3.6)
The sex category of the suspected loperamide abusers *	
○ Females	4 (4.8)
○ Males	50 (60.2)
○ Both	29 (34.9)
The age group of the suspected loperamide abusers *	
○ 20–30	48 (57.8)
○ 31–40	25 (30.1)
○ Variable	10 (12.1)
Type of customers who request loperamide for suspected abuse purposes *	
○ Strangers	14 (16.9)
○ Regular customers	1 (1.2)
○ Both	68 (81.9)
From your experience, the rate of abuse of loperamide is *	
○ Decreasing	3 (3.6)
○ No changes	9 (10.8)
○ Increasing	71 (85.5)
**What was the largest quantity (packs) of this product I have ordered for a single patient? ^$^**	
Mean (SD)	33.2 (14.9)
Pharmacists could recognize suspected loperamide abusers by (more than one answer was allowed) *	
○ Ask directly and acknowledge their needs	57 (68.7)
○ Unusual appearance (extreme lack of concern for appearance or dress)	62 (74.7)
○ Mood disturbances, inability to control impulses, and thought disturbances may appear	74 (89.2)
○ Exaggerates medical problems and/or mimics symptoms to obtain medication	75 (89.2)
○ Often orders medication specifically and refuses alternatives to other antidiarrheal drugs	44 (53.0)
○ Reluctance or unwillingness to provide reference information	60 (72.3)
○ May show extraordinary knowledge of antidiarrheals or controlled drugs	43 (51.8)
○ None, unrecognizable	2 (2.4)

^$^ The available packs in Jordan range from six to ten caps or tabs per pack. * Percentages were calculated based on the number of the pharmacists who reported loperamide abuse (n = 83).

**Table 3:** Logistic regression analysis for the identified independent variables associated with reported loperamide abuse among the study participants during the last six months.

Predictor	*p*-Value	Odds Ratio	95% CI	
			Lower	Upper
**Sex**				
○ Female	Ref			
○ Male	**0.001**	1.269	0.122	1.590
**Educational level**				
○ Diploma	Ref			
○ University degree	0.696	1.642	0.136	19.791
**Location of the pharmacy**				
○ Non-center of Jordan	Ref			
○ Center of Jordan (including the capital)	**0.005**	21.238	2.457	183.588
**Pharmacy location is in the**				
○ Main Road	Ref			
○ Side Road	0.069	22.193	0.790	623.318
**Years of experience in the pharmaceutical field**				
○ <10	Ref			
○ >10	0.590	0.7299	0.232	2.292
**Working shift at the pharmacy**				
○ Shift A/B	Ref			
○ Third Sift (C, 12–8 a.m.)	**0.002**	1.288	0.129	2.0842
**The general socioeconomic status of patients who visit the pharmacy**				
○ Middle/High	Ref			
○ Low/Low–Middle	0.062	0.230	0.049	1.079

Significance measure at *p* < 0.05 and presented in bold.

## Data Availability

Not applicable.
